# Hybrid Formation and Fusion of Cancer Cells In Vitro and In Vivo

**DOI:** 10.3390/cancers13174496

**Published:** 2021-09-06

**Authors:** Ralf Hass, Juliane von der Ohe, Thomas Dittmar

**Affiliations:** 1 Biochemistry and Tumor Biology Laboratory, Department of Obstetrics and Gynecology, Hannover Medical School, 30625 Hannover, Germany; Ohe.Juliane.von.der@mh-hannover.de; 2 Institute of Immunology, Center of Biomedical Education and Research (ZABF), Witten/Herdecke University, 58448 Witten, Germany

**Keywords:** hybrid cell formation, horizontal gene transfer/lateral gene transfer, mesenchymal stroma-/stem-like cells, heterokaryon-to-synkaryon transition, cell fusion

## Abstract

**Simple Summary:**

Cell fusion as a fundamental biological process is required for various physiological processes, including fertilization, placentation, myogenesis, osteoclastogenesis, and wound healing/tissue regeneration. However, cell fusion is also observed during pathophysiological processes like tumor development. Mesenchymal stroma/stem-like cells (MSC) which play an important role within the tumor microenvironment like other cell types such as macrophages can closely interact and hybridize with cancer cells. The formation of cancer hybrid cells can involve various different mechanisms whereby the genomic parts of the hybrid cells require rearrangement to form a new functional hybrid cell. The fusion of cancer cells with neighboring cell types may represent an important mechanism during tumor development since cancer hybrid cells are detectable in various tumor tissues. During this rare event with resulting genomic instability the cancer hybrid cells undergo a post-hybrid selection process (PHSP) to reorganize chromosomes of the two parental nuclei whereby the majority of the hybrid population undergoes cell death. The remaining cancer hybrid cells survive by displaying altered properties within the tumor tissue.

**Abstract:**

The generation of cancer hybrid cells by intra-tumoral cell fusion opens new avenues for tumor plasticity to develop cancer stem cells with altered properties, to escape from immune surveillance, to change metastatic behavior, and to broaden drug responsiveness/resistance. Genomic instability and chromosomal rearrangements in bi- or multinucleated aneuploid cancer hybrid cells contribute to these new functions. However, the significance of cell fusion in tumorigenesis is controversial with respect to the low frequency of cancer cell fusion events and a clonal advantage of surviving cancer hybrid cells following a post-hybrid selection process. This review highlights alternative processes of cancer hybrid cell development such as entosis, emperipolesis, cannibalism, therapy-induced polyploidization/endoreduplication, horizontal or lateral gene transfer, and focusses on the predominant mechanisms of cell fusion. Based upon new properties of cancer hybrid cells the arising clinical consequences of the subsequent tumor heterogeneity after cancer cell fusion represent a major therapeutic challenge.

## 1. Introduction

Cell fusion is a fundamental biological mechanism that describes the plasma membrane merging of two or more cells, thereby giving rise to one new hybrid cell displaying altered functionalities. Several physiological processes, such as fertilization, placentation, myogenesis, osteoclastogenesis, and wound healing/repair activities require the fusion of distinct somatic cells (for review see: [1,2,3,4,5,6,7,8,9]). Thereby, the formation of fused hybrid cells increases tissue plasticity. Moreover, the generation of new hybrid cell populations contributes to tissue homeostasis and elevates regenerative potential. However, cell fusion is also observed in a pathophysiological environment, including virus-infected host cells or during tumor development and subsequently evolving metastases (for review see: [1,3,4,5,6,7,8]).

Even though the process of cell fusion appears simple, like two soap bubbles merging, it is a tightly regulated, energy dependent, and not yet fully understood and characterized process. For instance, only certain cell types, such as cytotrophoblasts [10,11,12,13], myoblasts [14,15,16,17], or macrophages [9,18,19] possess fusogenic properties, whereby these cells are not per se fusogenic. To enable fusion the participating cells need to undergo a pre-hybrid preparation process (PHPP) accompanied by the formation of an associated hybrid-permissive environment. Accordingly, hybrid cells after cell fusion undergo a post-hybrid selection process (PHSP) to remodel DNA stability and to adapt to the normal cell metabolism [20,21].

But what is the fate of fusion-derived hybrid cells? Some of them remain in a stable multinucleated or so-called heterokaryon state, such as syncytiotrophoblasts, multinucleated muscle fibers, and osteoclasts [10,11,12,13,14,15,16,17,19,22,23,24]. In contrast, some bi- or multinucleated cells can also give rise to mononucleated daughter cells via a process that has been independently from the other named the “heterokaryon-to-synkaryon transition” (HST) [25] or synonymously “ploidy reductions” (PR) [26,27,28,29] and which has been observed in several cell types, including hepatic cells [26,27,28], hematopoietic cells [29], epithelial cells [30], and fibroblasts [31,32]. In accordance with the not yet fully understood process of cell fusion, the phenomenon of HST/PR also remains ambiguous.

The complexity of HST/PR is displayed by the transition of a cellular state carrying two discrete diploid nuclei from different parental cells to merge to a new functional nuclear unit with initially a tetraploid chromosomal phenotype. During this transition, the mis-segregation of the chromosomes from the two (or more) paternal nuclei can occur whereby HST/PR can lead to the induction of aneuploidy [27,33,34,35,36,37,38]. Finally, different mechanisms exist, such as entosis, emperipolesis, cannibalism, therapy-induced polyploidization/endoreduplication, and horizontal gene transfer/lateral gene transfer (HGT/LGT), which could also give rise to cell fusion like-derived cells.

## 2. Cell Fusion in General

Cell fusion is a tightly regulated process, which can be distinguished by an initiation and a termination phase. Zhou and Platt postulated that cell fusion can be subdivided into five discrete steps, including (i) priming, (ii) chemotaxis, (iii) adhesion, (iv) fusion, and (v) post-fusion [39]. This suggestion has been supplemented by further cellular programs including a PHPP and a PHSP [20,21] which are summarized in a hypothetic scheme. 

**Figure 1:**
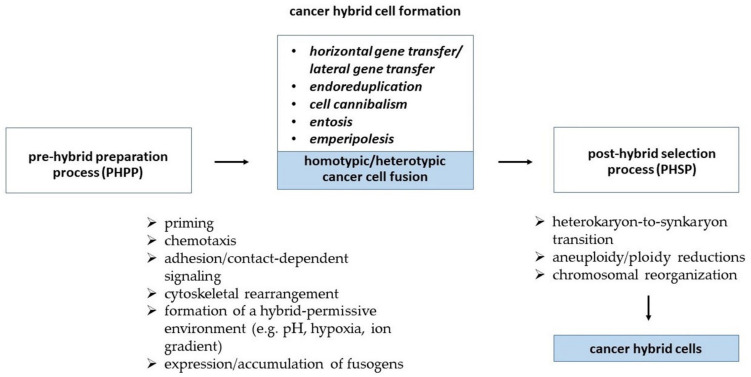
The formation of cancer hybrid cells is composed of an orchestrated sequence of distinct cellular programs which can be distinguished as (i) a pre-hybrid preparation process (PHPP), (ii) the cancer cell hybridization process, and (iii) a subsequent post-hybrid selection process (PHSP). Several events can lead to cancer cell hybridization including the fusion of cancer cells with neighboring cancer cells (homotypic fusion) or with other cell types (e.g., macrophages, cancer-associated fibroblasts (CAFs), MSC) within the tumor microenvironment (heterotypic fusion).

During the priming step cells acquire the ability to fuse, which is paralleled by an expression of adhesion molecules and alterations in the lipid composition of the plasma membranes. These changes are performed by a translocation of inner-leaflet lipids and the loss of an inhibitory state as a consequence of extracellular matrix degradation allowing facilitated cell migration and close cell–cell contact as a physical requirement of cell fusion [39]. The fusion step represents a complex orchestration of thermodynamic and biochemical processes which are yet not fully understood. Fusion-specific/-related proteins termed fusogens (for reviews see: [4,6,8,40]) play a pivotal role in overcoming certain energetic barriers. In particular, fusogens are mandatory for the contribution to steric formation of several cell fusion-related lipid intermediates named “the hallmarks of cell–cell fusion” [4]. These include (i) dehydration (thereby bringing phospholipid heads of the two cellular fusion partners in close vicinity of less than one nanometer), (ii) merging of the outer monolayers of hemifusion via a stalk and/or diaphragm intermediate, and (iii) opening and expansion of fusion pore(s) from nanometer diameters to multiple microns to enable fusion [4]. Finally, once the cells have merged they enter a post-hybrid state and become non-fusogenic again. In accordance with the still unresolved complexity of the fusion step it remains unclear which inter- and intracellular signaling pathways are involved in turning a cell from a fusogenic into a non-fusogenic state (Figure 1).

Well-known fusogens in physiological human processes are syncytin-1 and syncytin-2, which are involved in the fusion of villous cytotrophoblasts to multinucleated syncytiotrophoblasts [13,41,42,43,44], myomaker and myomerger, that play a role in myogenesis [45,46,47,48] and Izumo1 (sperm) and Juno (oocyte), which are crucial for fertilization [49,50,51,52]. In contrast, despite the fact that several proteins that are involved in macrophage fusion, such as CD9, CD47, CD81, CD200, DC-STAMP, OC-STAMP, and MMP9 (for review see: [1,7,9]), no macrophage specific fusogens have been identified so far. Interestingly, syncytin-1 expression was also found in differentiating osteoclasts suggesting an involvement of this fusogen in osteoclastogenesis [22,53].

In addition to fusogens it is well known that cell fusion also depends on the reorganization of the actin cytoskeleton and crosstalk with plasma membrane phospholipids, in particular phosphatidylserine (PS), PS-binding annexins, and further membrane proteins (Figure 1) (for review see: [1,4,8,39,54,55,56,57,58,59]). Notably, PS signaling has been suggested as a uniquely conserved signaling module in cell–cell fusion, as PS exposure in the outer leaflet of plasma membranes has been found in myoblasts (myogenesis), macrophages (osteoclastogenesis), trophoblasts (placentation), sperm (fertilization), and even cancer cells (for review see: [57]). The translocation of PS from the inner leaflet to the outer leaflet of the plasma membrane is facilitated by the large family of professional phospholipid scramblases via at least three independent pathways, such as Ca^2+^-activated phospholipid scrambling (Ca^2+^-PLS), caspase-activated phospholipid scrambling (Cas-PLS), and constitutive phospholipid scrambling (for review see: [57]). Indeed, studies have revealed the involvement of transmembrane member 16 (TMEM16) Ca^2+^-PLSases family members in myogenesis [60,61], osteoclastogenesis [62], and placentation [63]. Anoctamin 5/TMEM16E knockout mice (*Ano5^−/−^*) muscle progenitor cells (MPCs) exhibited defective cell fusion in culture and produced muscle fibers with significantly fewer nuclei compared with controls, which could be corrected by the viral introduction of the *ANO5* gene in *Ano5^−/−^*MPCs [60]. Likewise, fusion among the human choriocarcinoma trophoblastic BeWoc cell line was abolished by the genetic ablation of the TMEM16F Ca^2+^-PLSase and the placentas of TMEM16F-deficient mice exhibited deficiencies in trophoblast fusion and fetal blood vessel development [63]. How PS facilitates cell–cell fusion is not yet clear. As excellently reviewed by Whitlock and Chernomordik, PS could be either recognized by cells expressing PS receptors and/or PS binding proteins, and/or functions at the remodeling stage of cell fusion [57]. For instance, data from Hochreiter-Hufford and colleagues and Park et al. revealed that the fusion of myoblasts was mediated by PS-exposing apoptotic cells via the PS receptor BAI1 and associated protein complexes with ELMO and Dock180 [64,65]. Interestingly, apoptotic cells did not directly fuse with the healthy myoblasts, but rather induced a contact-dependent signaling with neighbors to promote fusion among the healthy myoblasts [64]. Likewise, the PS receptor CD36 facilitates the fusion of myoblasts [66], macrophages [67], and cancer cells [68]. Data from Verma and colleagues revealed that the inhibition of TMEM16 PLSases or the perturbation of the expression or activity of annexins A1 and A5 markedly impaired the fusion between osteoclast precursors, suggesting that PS exposure played a role in the membrane remodeling stages of cell fusion [62].

Regeneration studies of bone marrow-derived cells (BMDC) have revealed an elevated frequency of cell fusion events in inflamed tissues as compared to a non-inflammatory environment [69,70,71], indicating that inflammation can trigger cell fusion. This assumption is supported by the findings of the pro-inflammatory cytokine tumor necrosis factor-α (TNF-α) to increase fusion and osteoclastogenesis [72]. Further evidences have been provided by several studies also in a pathophysiological environment demonstrating enhanced rates of fusion events between cancer cells and normal cells, including epithelial cells, endothelial cells, or mesenchymal stroma/stem-like cells (MSC) in the presence of TNF-α [72,73,74,75,76,77,78]. Alternative to these processes, several studies have revealed that cell fusion can also occur in the absence of inflammation [29,69,70] adding to the complexity of the entire phenomenon.

In summary, the process of cell fusion and how it is initiated and terminated is still only scarcely understood. Fusogenic proteins in combination with cytoskeletal reorganization which can be induced by inflammation/inflammatory cytokines and apoptosis can support cell fusion, e.g., by converting cells from a non-fusogenic into a fusogenic state or by increasing cell–cell contacts (Figure 1). In this context, a pro-inflammatory environment generated by viral or bacterial infection can also contribute to cell fusion and neoplastic development. 

## 3. DNA Reorganization during Cancer Cell Fusion

### 3.1. DNA Transfer by Horizontal or Lateral Gene Transfer

Horizontal gene transfer (HGT), also known as lateral gene transfer (LGT), is the transfer of genetic material between unicellular and multicellular organisms of all living matter and has been suggested as an important factor in the evolution of many organisms [79,80,81]. Thereby, DNA as well as RNAs (mRNA and microRNA) may be transferred by different mechanisms, such as (1) transduction (including viruses and bacteriophages), (2) transformation (of cell-free DNA), (3) conjugation (via F-pili), (4) gene transfer agents, (5) nanotubes, and (6) extracellular vesicles (EVs, such as apoptotic bodies, microvesicles, and exosomes) [79,80,81]. In this connection, it is well known that HGT/LGT is one of the driving mechanisms for the rapid spread of multidrug resistance bacteria worldwide [82]. 

Even though HGT/LGT is best studied in bacteria, fungi, insects, and plants, this process also occurs in mammals, whereby genetic material is predominantly transferred by extracellular vesicles and even cell-free DNA (for review see: [80,83]). However, with regard to the size of extracellular vesicles and the underlying mechanisms that will result in the release of cell-free DNA, it can be concluded that not intact chromosomes, but rather DNA fragments and even circular DNA, will be transferred. Thereby, the size of cell-free DNA could vary between 166 bp to 498 bp of apoptotic-derived nucleosomes and polynucleosomes and up to 20,000 bp of extrachromosomal circular DNA [83]. Indeed, some studies have revealed normal murine NIH 3T3 fibroblasts to be transformed by HGT/LGT due to the uptake of tumor cell-derived cell-free DNA harboring oncogenes [84,85,86]. 

In addition to cell-free DNA, it is well recognized that DNA, mRNAs, miRNAs, and proteins are transferred via extracellular vesicles, including apoptotic bodies, microvesicles, and exosomes (for review see: [80,83]). In accordance with cell-free DNA, whole genes can be transferred via apoptotic bodies, which are functionally integrated into the recipient cell genome. For instance, Bergsmedh and colleagues showed that DNA encapsulated in apoptotic bodies derived from H-rasV12- and human c-myc-transfected rat fibroblasts was found in the nuclei of recipient murine cells, which was further associated with a loss of contact inhibition in vitro and a tumorigenic phenotype in vivo [87]. Likewise, the co-cultivation of cell lines containing integrated copies of Epstein-Barr virus (EBV) resulted in a rapid uptake and transfer of EBV-DNA as well as genomic DNA to the nucleus of the phagocyting cells [88]. Moreover, data from Ehnfors and colleagues revealed that both fibroblasts and endothelial cells are capable of acquiring and replicating DNA derived from apoptotic SV40 large T antigen positive tumor cells [89]. In vivo data demonstrated that xenotransplanted tumors in severe combined immunodeficient mice exhibited a sub-population of endothelial cells containing tumor DNA, which maintained the ability of vessel formation and concurrently expressed tumor-encoded endothelial-specific genes [89]. With regard to cell fusion, a study of de la Taille et al. demonstrated that the co-cultivation of neomycin-resistant LNCaP prostate cancer cells with hygromycin-resistant LNCaP (LNCaP-HygR) cells resulted in the origin of dual antibiotic resistant cells after the induction of apoptosis in LNCaP-HygR cells [84]. The dual antibiotic selection assay is commonly used for the generation and selection of cancer hybrid cells. Hence, cancer hybrids have to be further validated by additional methods, such as short tandem repeat analysis, to exclude the possibility of HGT/LGT-mediated dual antibiotic resistance.

### 3.2. Role of Extracellular Vesicles (EVs) in Cancer Cell Fusion 

EVs are well-known carriers for DNA, various types of RNA, lipids, and proteins, playing an essential role in intercellular communication and the regulation of diverse physiological and pathophysiological processes (for review see: [80,90,91,92]). In contrast to apoptotic bodies, microvesicles (50 to 500 nm) and exosomes (50 to 150 nm) are much smaller and are actively secreted by cells (for review see: [90,91,92]). Thereby, microvesicles are formed by budding of the plasma membrane, whereas exosomes are formed as intraluminal vesicles from multivesicular endosomes that subsequently fuse with the plasma membrane to release their content (for review see: [90,91,92]). 

Microvesicles and exosomes, in addition to apoptotic bodies, can transfer genomic donor DNA to recipient cells, whereby double stranded DNA fragments of up to 17,000 bp have been detected [93,94,95,96]. For instance, exosomes isolated from the peripheral blood of chemotherapy-treated melanoma patients contained double stranded “self-DNA” derived from intestinal tissue, which were internalized by macrophages and dendritic cells [96]. The release of “self-DNA” into the cytoplasm activated the melanoma 2 (AIM2) inflammasome concomitant with the secretion of the pro-inflammatory cytokines IL-1β and IL-18 [96]. 

High copy numbers of Arabidopsis thaliana-DNA (A.t.-DNA) were detected in EVs derived from lentivirally A.t.-DNA-transduced human bone marrow MSCs [95]. A thorough analysis of recipient cells treated with these extracellular vesicles finally revealed a stable integration of A.t.-DNA in the cells’ genome, indicating that HGT/LGT could be mediated by EVs [95]. 

These findings are also in line with data from Cai and colleagues demonstrating that AT_1_-DNA and the BCR/ABL hybrid gene were found in EVs and could be transferred to HEK293 cells and neutrophils [93,94]. Additional findings revealed the in vivo relevance of EV-mediated DNA transfer, since BCR/ABL DNAs-containing EVs display pathophysiological characteristics of CML, such as feeble, febrile, splenomegaly, and neutrophilia two months after injection [94]. 

In addition to transfer of biological materials, EVs have been associated with the process of cell fusion. Whether EVs can directly mediate cell–cell fusion by acting as a linker to bridge two individual cells remains ambiguous. In any case, exosomes released by trophoblasts carry molecules involved in placental physiology, including syncytins as fusogens [97]. These fusogenic factors include sequestered or truncated genes originating from viral functions such as the HERV-W retroviral envelope genes that have been domesticated in the mammalian genome. Accordingly, EVs can carried syncytin-1 [98] and may contribute to cancer cell fusion [99]. 

## 4. Cell Cannibalism, Entosis, and Emperipolesis 

Alternative processes of cell merging predominantly observed in cancer cells include cell cannibalism, entosis, and emperipolesis (Figure 1).

During cell fusion various cell types can hybridize mediated by different fusogens and the resulting combined plasma membranes give rise to a bi- or multinucleated heterokaryon with a mixed cytoplasm. In contrast, cell cannibalism, entosis, and emperipolesis of cancer cells are among those so-called cell-in-cell phenomena (for review see: [100,101,102,103,104,105,106]). 

### 4.1. Cell Cannibalism

Cell cannibalism displays similarities to phagocytosis (Figure 1). However, in addition to the engulfment of cell debris and apoptotic cells/bodies, cannibalistic cancer cells also have the capability to actively “phagocytose” living cells, such as other cancer cells (homotypic cannibalism) or leukocytes/lymphocytes and MSCs (heterotypic cannibalism). This process is supported by factors including the vacuolar-type ATPase proton pump-activating transmembrane 9 superfamily 4 protein TM9SF4, the plasma membrane and actin cytoskeleton linker protein Ezrin (cytovillin, villin-2), and the integrin subunits to tyrosine kinases linker and scaffolding protein caveolin-1, usually resulting in a complete lysosomal digestion of the cannibalized cells [100,105,107,108,109,110,111,112,113,114]. It is commonly assumed that the reason for cannibalism among cancer cells is the lack of nutrition in proliferating neoplastic tissues whereby cannibalizing other living cells appears to be the only alternative for cancer cells to survive in this environment [113,115,116]. In addition, cannibalism might also play a role in the immune escape of cancer cells [117,118]. 

### 4.2. Entosis and Emperipolesis

**Figure 2:**
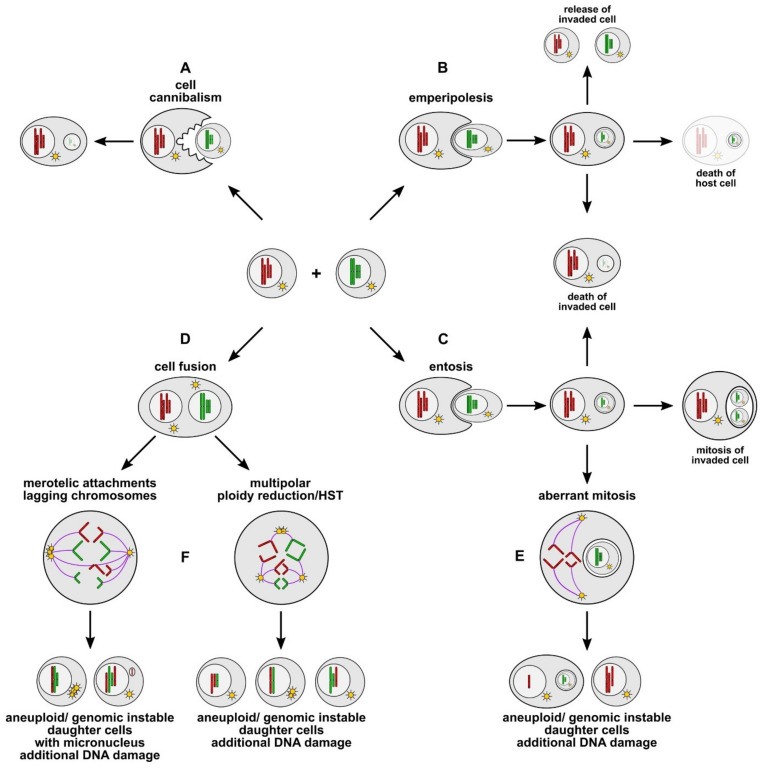
Different fates of merged cells. Cell cannibalism (**A**), emperipolesis (**B**), and entosis (**C**) belong to the so-called cell-in-cell phenomena, which are characterized by the engulfment of intact cells. In contrast, cell fusion (**D**) gives rise to bi- or multinucleated heterokaryons due to merging of the cells’ plasma membranes. Cell cannibalism (**A**) resembles “phagocytosis” and usually results in the lysosomal digestion of the engulfed cell. In contrast, in emperipolesis (**B**) and entosis (**C**) one cell actively invades another cell, whereby the fate of the engulfed cell differs markedly between processes. Emperipolesis is characterized by cells either escaping from the host cell, being destroyed by the host cell, or vice versa destroying the host cell (**B**). On the contrary, most entotic cells are usually destroyed by lysosomal degradation, whereas some internalized cells may also survive and can even divide within the host cell (**C**). Moreover, entosis may be associated with aberrant mitosis (**E**) and the origin of aneuploid and genomic instable daughter cells. A characteristic of hybrid cells is the merging of parental chromosomes and their random segregation to daughter cells during bi- and multipolar divisions (**F**), which is also associated with aneuploidy, genomic instability, and micronucleus formation.

In contrast to cell engulfment and subsequent digestion in cannibalism, cells actively invade host cells in entosis and emperipolesis, thereby building a cell-in-cell structure (Figure 1 and Figure 2; for review see: [100,101,102,104]). Although both phenomena display morphological similarities, some functional differences have been characterized. Emperipolesis is observed mostly in cells of hematopoietic origin, such as lymphocytes or NK cells, by invading into host cells like megakaryocytes, thymic nurse cells, liver cells, or cancer cells [100,102,119,120]. Proteins like Ezrin, the leukocyte function-associated antigen-1 integrin LFA-1, and its ligand the intercellular adhesion molecule-1 ICAM-1, contribute to emperipolesis. Different possible outcomes accompany emperipolesis: invaded cells can either escape from the host cell, or may be destroyed by the host cell, or vice versa may destroy the host cell [100,102] (Figure 2). 

In entosis, internalized cells such as cancer or epithelial cells (named “loser cells”) actively invade other epithelial or cancer cells (named “winner cells”) [100,102]. Entosis depends on various factors such as glucose, E- or P-Cadherin, the G-Protein Rho A and the associated kinase ROCK, and the AMP-activated protein kinase (AMPK). Compared to emperipolesis, the fates of entosis-internalized cells are different [100,102,121]. While most entotic cells are usually destroyed [122] by lysosomal degradation, some internalized cells can survive and even divide within host cells [101,102] (Figure 2).

Similar to cell fusion, the entosis of cancer cells is associated with the induction of aneuploidy and chromosomal instability, although via different effects. The entotic cell acts as a barrier, which leads to a failed cytokinesis in its host cell [100,101,102,106,121]. Whether entosis can develop a hybrid cell as occurs after fusion remains to be elucidated. This would require a recombination of the internalized cell genome with the nucleus of the host cell. Data from Sottile et al. revealed that the co-cultivation of embryonic stem cells (ESCs) and MSCs results in both cell fusion-derived hybrids and entotic hybrids, which were able to proliferate and to form ESC- and MSC-like colonies [123]. In contrast to cell fusion-derived hybrids undergoing HST/PR, however, entotic cells were either released or degraded [123]. Similar effects were observed in co-cultivation experiments using human MSCs and human U87 glioblastoma cells whereby entotic cells were degraded [124]. While entosis was also observed in co-cultivation experiments using human breast cancer cell lines, little is known about the fate of entotic cells [125].

## 5. Cell Fusion in Tumors 

The hypothesis that cell fusion might play a role in tumor development had already been postulated by the German physician Otto Aichel more than 100 years ago in 1911 [126]. Aichel assumed that the aneuploidy of cancer cells and leukocyte-like properties of metastatic tumors were attributed to fusion events between tumor-invading leukocytes and cancer cells [126]. Since then, a plethora of in vitro and in vivo studies on human tumors have been published supporting Aichel’s hypothesis that cancer cells are fusogenic. Moreover, hybridization with other cancer cells or with adjacent non-tumor cell types, including macrophages, fibroblasts, and stem cells have generated new cancer hybrid cells with increased metastatic potential and more chemotherapy and radiotherapy resistant properties than parental cancer cells [32,73,123,125,127,128,129,130,131,132,133,134,135,136,137,138,139,140,141,142,143,144,145,146,147,148,149,150,151,152,153,154,155,156,157,158,159,160,161,162,163,164,165,166,167,168,169,170,171,172,173,174,175,176,177,178,179,180,181,182,183,184,185,186,187,188,189,190,191,192,193,194,195,196,197,198,199]. Consequently, tumor progression could be fostered due to the generation of more metastatic tumor hybrids, which can evade from the primary tumor and form secondary lesions both in the lymph nodes and in distant organs. Likewise, chemotherapy and/or radiotherapy resistant tumor hybrids may survive cancer therapy and become the seeds for recurrences. Appropriate examples are given in the following subchapters (see below). Additionally, cell–cell fusion has been suggested as a driver in tumor heterogeneity, which is related to genomic instability and a hallmark of solid tumors [200]. Assuming a fusion probability of 6.6 × 10^−3^ in vitro and 6.6 × 10^−5^ in vivo together with values of genetic mutation rates of 10^−6^ to 10^−3^ as well as the number of potential driver genes of about 300 mathematical calculations revealed a substantially enhanced increase in clonal richness and overall mutation rate in a 3D environment as compared to only “mutated” cells [125]. Hence, despite a relatively low frequency of spontaneous somatic cell–cell fusion events and the impact of spatial constraint, fusion-mediated recombination could have a profound impact on somatic evolution through the accelerated diversification of tumor cell populations and the generation of rare mutational variants capable of exploring larger swathes of adaptive landscapes [125]. Such cell–cell fusion derived populations could then be the seeds for metastatic lesions and/or could be resistant to chemo- and/or radiotherapy.

How cancer cells fuse is still not clear, but it is most likely to be similar to the fusion of normal cells and facilitated by fusogens, such as syncytin-1 [77,98,168,201,202,203], PS and PS-binding proteins [98,136], and inflammation/inflammatory cytokines [76,77]. Likewise, tumor cells are not per se fusogenic, but have to be transferred into a fusogenic state first. In this connection, the co-cultivation of human prostate cancer cells with muscle cells resulted in an IL-4 and IL-13 dependent upregulation of syncytin-1 and annexin 5 expression and enhanced frequency of homotypic cancer cell–cell fusion [98] suggesting that both cytokines might be involved in the conversion towards a pro-fusogenic state of (cancer) cells. This would be in view with findings showing that the exposure of macrophages to IL-4 and IL-13 induced homokaryon formation [9]. However, the detection of cancer hybrids in vitro and in vivo is challenging and depends on valid and reliable fusion markers since other multinucleoli- or aneuploidy-generated mechanisms such as entosis, emperipolesis, cannibalism, therapy-induced polyploidization/endoreduplication, and HGT/LGT could also give rise to cancer hybrid-like cells.

### 5.1. Evidence for Cancer Cell Fusion by In Vitro Studies

Several studies using genetically modified cancer cells and transgenic mouse models have revealed cancer cell fusion among themselves (homotypic fusion) or with other cell types in the tumor microenvironment, such as macrophages, CAFs, or MSC (heterotypic fusion) [31,32,73,123,125,127,128,129,130,131,132,133,134,135,136,137,138,139,140,141,142,143,144,145,146,147,148,149,150,151,152,153,154,155,156,157,158,159,160,161,162,163,164,165,166,167,168,169,170,171,172,173,174,175,176,177,178,198,204,205,206]. 

Markers to identify cancer cell fusion and to isolate cancer hybrid cells include resistance to different antibiotics, or the co-expression of two different fluorescent reporter proteins [31,32,73,123,128,129,133,134,137,138,140,141,142,143,144,145,147,148,149,150,151,152,154,155,158,161,162,164,167,169,171,172,173,174,178], or the use of hypoxanthine, aminopterin, or thymidine (HAT) medium [125,160,173,175], respectively. 

In more detail, a variety of different cell systems provide evidence for the acquisition of fusion-derived new properties in cancer hybrid cells, e.g., the co-cultivation of bone- and lung-tropic sublines of the human MDA-MB-231 breast cancer cell line with resistance to either puromycin or hygromycin in media supplemented with both antibiotics resulted in the appearance of double resistant hybrid cells exhibiting a dual metastatic organotropism [158]. Likewise, a dual antibiotic selection strategy was used for the generation of hybrid cells derived from spontaneous fusion events between human breast epithelial cells and human breast cancer cells [128,129,178]. Functional changes were also observed in M13HS-2 and M13HS-8 hybrid cells that were derived after the fusion of human M13SV1-EGFP-Neo breast epithelial cells with human HS578T-Hyg breast cancer cells. These cancer hybrid cells responded to the chemokine CCL21 with an increased migratory activity, whereas the parental cells did not [127]. Since CCL21 has been associated with lymph node metastases in breast cancer [207], these findings likely indicate that the fusion of CCL21-insensitive cancer cells could give rise to CCL21-sensitive cancer hybrid cells. In addition, M13HS hybrids display certain cancer stem/initiating cell properties, such as an increased mammosphere and colony formation capacity as well as elevated ALDH1 expression [208]. This is in line with previous suggestions that cancer stem/initiating cell properties may be acquired during cell fusion events [178]. 

A dual antibiotic selection/fluorescence reporter strategy has also been used to isolate hybrid cells derived from lung IMR90 E6 E7 H-RAS_G12V_ CFP-Blast cells and IMR90 E6 E7 SmallT hTERT DsRed-Puro cells [32]. The aim of this study was to prove whether the fusion-mediated oncogenic combination of hTERT, SV40 SmallT antigen, and H-RAS_G12V_ would result in a malignant conversion of non-transformed immortalized fibroblasts. Indeed, hybrids harboring all five oncogenes not only exhibited an increased and aneuploid mean chromosomal number but were also highly tumorigenic [32]. In a similar study, cancer hybrid cells derived from neoplastic IMR90 E6E7 H-RAS_G12V_ SmallT hTERT DsRed PuroR and non-transformed IMR90 E6E7 CFP BlastR cells were selected based on their co-resistance to blasticidin and puromycin [172]. These hybrids were highly aneuploid and revealed non-recurrent, large-scale genomic rearrangements including interchromosomal translocations [172]. 

In a sarcoma study, cancer hybrids exhibited novel phenotypic traits such as metastatic spreading capabilities and were the only cells that recapitulated in vivo all features of pleomorphic sarcomas [172]. Similar findings were reported for hybrid cells that were derived from immortalized myoblasts and transformed fibroblasts by exhibiting clonogenic ability and dissemination properties [31]. Moreover, hybrid tumors were found to mimic the histological characteristics of undifferentiated pleomorphic sarcomas with incomplete muscular differentiation, suggesting that cell fusion might favor specific sarcoma development according to the differentiation lineage of parent cells [31]. 

The co-cultivation of RFP-tagged G418 resistant LNCaP prostate cancer cells with GFP-labeled puromycin-resistant prostate stromal cells resulted in the origin of RFP/GFP double positive and dual antibiotic-resistant hybrids displaying divergent behaviors and exhibiting permanent genomic hybridization [142]. Lindstrom and colleagues co-cultivated human M2-macrophages and GFP-labeled MCF-7 human breast cancer cells and spontaneously formed GFP^+^, CD163^+^, and CD45^+^ tumor hybrids were sorted by flow cytometry [132]. After treatment with γ-radiation, tumor hybrids showed an increased survival fraction and colony formation ability, which was accompanied by an overall lesser DNA damage as compared to parental MCF-7 breast cancer cells [132] indicating that cell–cell fusion could give rise to radioresistant cells.

### 5.2. Cancer Cell Fusion as a Rapid Process and In Vivo Studies

Previous work has demonstrated that RFP-labeled omental adipose-derived stromal (O-ASC) cells spontaneously fused with GFP-marked endometrial cancer cells (ECC), whereby the resulting cancer hybrid cells displayed a mesenchymal phenotype [169]. These hybrids proliferated through bipolar and multipolar divisions and showed an increased migratory capacity, suggesting epithelial-to-mesenchymal-associated changes including the down-modulation of E-cadherin and the up-regulation of Vimentin [169]. Time-lapse video microscopy could verify the direct fusion of RFP-O-ASCs with GFP-ECCs [169]. Similar morphological documentations were provided by Melzer and colleagues demonstrating that the fusion of GFP-tagged MSCs with mCherry-tagged MCF10A non-tumorigenic breast epithelial cells or mCherry-tagged human mammary epithelial cells (HMECs) occurred within less than five minutes [73]. 

In vivo studies have revealed a significantly elevated tumor growth of human breast cancer hybrid populations (MDA-MSC-hyb1 and –hyb2) together with the development of multiple distant organ metastases in a much shorter period of time than the parental breast cancer cells [153]. Similar findings have been reported by Gast and colleagues who demonstrated the spontaneous fusion of GFP-tagged murine macrophages with H2B-RFP-labeled MC38 mouse colon carcinoma cells, thereby giving rise to highly motile and metastatic hybrids [164]. Time-lapse movie data substantiated this fusion of H2B-RFP MC38 cells and a GFP-tagged macrophage with the subsequent division of the evolving hybrid cell into two daughter cells [164]. An altered tumor behavior was observed in MDA-MSC-hyb5 breast cancer hybrid populations displaying an initial tumor dormancy. Following tumor initiation after dormancy, however, tumor growth and the formation of various different organ metastases occurred much faster as compared to the parental breast cancer cells [209]. A Cre-LoxP recombination strategy has been used in additional studies either to visualize cell fusion [166,198] or to quantify cell fusion events between human breast epithelial cells and human breast cancer cell lines [177]. Beside these cell fusion strategies, the further hybridization of cancer cells and normal cells was also visualized by bimolecular fluorescence complementation (BiFC) [136], dual split reporter assays [210], co-expression of lineage specific surface markers [138,164,165,191,192,193,194], or karyotypization [131,156,165,187].

Although these data support the occurrence of homotypic and heterotypic cancer cell fusion events in vitro and in vivo, further characterization and distinction of cancer hybrid cells require karyotypization, short tandem repeat (STR) analysis, or FISH analysis. This, however, is limited in cancer hybrid cells of homotypic fusions originating from parental cells with an identical genomic background [211]. Thus, it cannot be ruled out that the acquisition of parental genes was mediated by HGT/LGT due to the up-take of apoptotic bodies or extracellular vesicles such as exosomes rather than by cell fusion [87,89,212]. 

### 5.3. Indirect Evidence for Cell Fusion by Clinical Observations of Hybrid Cells in Human Tumors 

Several in vitro and in vivo animal studies have demonstrated that cancer cells exhibit fusogenic properties and may either homo- or hetero-hybridize with the generation of new cancer hybrid cells displaying altered properties [138,164,180,181,182,183,184,185,186,187,188,189,190,191,192,193,194,195,196,197].

A common approach to search for putative heterotypic fusion is to seek the expression of non-cancer specific epitopes in cancer hybrid cells, such as macrophage, hematopoietic, or epithelial antigens [138,182,183,184,191,194]. However, the expression of hematopoietic and epithelial lineage markers of cancer cells can also be traced back to genomic instability, which limits this strategy.

In any case, Shabo and colleagues detected the expression of macrophage antigens DAP12, MAC387, and CD163 in human breast cancer and colorectal cancer tissues [182,183,184,197]. Increased CD163 expression levels were related to early recurrence and reduced overall survival of afflicted patients [183,184], suggesting CD163 as a suitable prognostic factor. 

Putative cell fusion-derived cancer hybrid cells co-expressing EpCAM/CD45 and CD125/CD45, respectively, were found in the ascites of ovarian carcinoma patients [138]. About 50% of these cancer hybrid cells also expressed the promigratory chemokine receptor CXCR4 [138], suggesting an acquired metastatic capacity. The expression of CD117 and CD44, which has been associated with a cancer stem cell phenotype in ovarian cancer, was found in up to 90% of EpCAM^+^/CD45^+^ ovarian cancer hybrid cells [138] indicating an association of cell fusion with the origin of cancer stem/initiating cells. Indeed, cell fusion has been proposed as a potential mechanism that could give rise to cancer stem/initiating cells [25,128,146,213].

Alternatively to the detection of non-cancer specific epitopes in cancer hybrid cells the identification of cancer-specific oncogenes/markers in non-cancerous cells represents a further strategy to identify putative cancer hybrid cells in vivo, e.g., a melanoma-derived mutated BRAF^V600E^ oncoprotein was detectable not only among cells surrounding the primary tumor but was also present in the stroma of melanoma metastases as well as in a histologically tumor-free re-excision sample from a patient who subsequently developed a local recurrence [190]. 

In addition to the identification within solid tumor tissues, cancer hybrid cells were also detectable in the circulation of cancer patients. Thus, CK^+^/CD45^+^ cancer hybrid cells were identified by gradient centrifugation in the peripheral blood of patients with melanoma, pancreatic, and colorectal cancer [191]. Thereby, cultured circulating melanoma-derived cancer hybrid cells concomitantly expressed macrophage markers (CD14, CD68, CD163, CD204, and CD206), melanocyte-specific markers (ALCAM, MLANA, and melanoma-specific BRAF^V600E^ mutations), epithelial cell markers (CK, EpCAM), and some stem cell-related markers (CXCR4, CD44) [192]. Similar findings were obtained with cultured circulating pancreatic cancer hybrid cells derived from pancreatic ductal adenocarcinoma patients [193]. Such cells were characterized by a co-expression of pancreatic cancer (stem cell) markers (CD44, ALDH1A1, CXCR4, ZG16B, S100PBP) and macrophage markers (CD204, CD206) [193]. Moreover, metastatic spreading along multiple tissues, including liver, spleen, and lung, was observed for cultured circulating pancreatic cancer hybrid cells following orthotropic transplantation into nude mice [193]. Rather, micro-metastases consisting of single cells or small groups of cells were formed within the organs, potentially serving as “niches” for subsequent colonization by metastasis-initiating cells [193]. Supportive findings were observed for non-small cell lung cancer (NSCLC). Thus, Manjunath et al. isolated circulating EpCAM^+^/CK^+^/CD14^+^/CD45^+^ cancer hybrid cells from the peripheral blood of NSCLC patients by immunomagnetic separation [194]. Circulating cancer hybrid cells were identified in 76.5% of NSCLC patients and the number of cancer hybrid cells in the circulation was correlated with AJCC tumor stages [194]. In addition to the overall number of circulating cancer hybrid cells, the authors also identified some giant circulating cancer hybrid cells with a diameter of more than 50 µm. These findings were associated with a significantly shorter overall and cancer-specific disease-free survival after curative surgery for stages I–IIIA and, hence, might be used as an independent survival predictor [194]. The appearance of multi-nucleated giant cells was also observed during the fusion of HIV-infected T cells with macrophages evolving virus-productive multinucleated immune hybrid cells [214]. Moreover, the fusion of cancer cells with leukocytes such as macrophages also contributes to increased tumor heterogeneity [164].

In sum, these data indicate that cancer cells expressing non-cancer specific epitopes (e.g., from macrophages, MSCs, or epithelial cells) were identified in human cancers indicating an origination of former cancer cell fusion events [132,133,137,156,160,164,165,215]. 

### 5.4. Direct Evidence for Cell Fusion by Clinical Observations of Hybrid Cells in Human Tumors 

Besides the limitations of lineage markers as indicators for cell fusion, some studies have focused on cancer patients with a former bone marrow transplantation (BMT) history or have specifically searched for tumor-specific DNA in commonly normal cells [164,185,186,188,189,196,216]. A combination of immunohistochemistry and FISH analysis in patient samples identified B-cell lymphoma-specific chromosomal translocations in 15 to 85% of microvascular endothelial cells, likely indicating that such cells originated from cell fusion [216]. In myeloma patients FISH analysis of bone-resorbing osteoclasts revealed that more than 30% of these multinucleated cells contained nuclei with translocated chromosomes of myeloma B-cell clone origin [187]. Moreover, these nuclei were fully integrated amongst the nuclei and were transcriptionally active [187]. Additional findings suggested that the multiple myeloma-induced disruption of bone remodeling compartments resulted in osteolytic lesions and provided a fusion permissive environment enabling the generation of bone-destructive osteoclast-myeloma hybrid cells [188]. 

The to-date gold standard for identifying cancer cell fusion in human tumors represents a mixed genomic phenotype of donor and recipient DNA in cancer hybrid cells from patients with a former BMT. Unfortunately, the number of such cancer patients is pretty rare. An analysis of a renal cell carcinoma metastasis for BMT DNA (recipient blood group A+; donor blood group O+) revealed that the tumor genotype was A/O indicating that such cells have been originated by cell fusion [189]. Likewise, donor Y chromosome was found in the renal carcinoma cells of a female BMT patient also suggesting that these cells were generated from hybridization events between “recipient” cancer cells and “donor” bone marrow-derived cells [196]. Similar findings were reported by Gast et al. demonstrating that Y chromosome-positive cancer cells were identified in a variety of different tumors, such as pancreatic ductal adenocarcinoma (PDAC), lung cancer, renal cell carcinoma, and head and neck squamous carcinoma (HNSCC) in women with previous sex-mismatch BMT [164]. In addition, EpCAM, CD45, and Y chromosome-positive circulating cancer hybrid cells were detected in PDAC patients whereby high cell numbers were correlated with a poor survival [164]. These results are in agreement with the overall assumption that cancer hybrid cells can acquire novel properties such as an altered metastatic behavior and a modified chemoresistance [217]. Strong evidences for cell fusion-derived cancer hybrid cells in human tumors were also provided by Lazova et al. and LaBerge et al. [185,186,218]. In both studies laser microdissected tumor hybrid cells from cancer biopsies were investigated by short tandem repeat (STR) analysis and revealed an overlap of different donor and recipient alleles [185,186]. Moreover, in both studies cancer hybrid cells were found in metastases, suggesting that the hybridization between donor and patient cells contributed to the promotion of secondary lesions [185,186]. A brief summary of the identification of tumor hybrid cells in human cancers is given in Table 1.

## 6. Conclusions

Besides several distinct mechanisms of cancer hybrid cell formation the development of cell fusion can significantly contribute to tumor heterogeneity and the generation of distal metastases. After preparation of cell fusion by PHPP and following fusion the surviving cancer hybrid cells during clonal convergence of a subsequent PHSP display altered functionalities. Consequently, the initially rare cancer hybrid cells may eventually develop a selection advantage to overgrow other cancer cells and contribute to increased tumor plasticity. Thus, cancer hybrid cells can eventually represent a prominent population within the tumor tissue to influence further tumor growth and to support enhanced metastatic diversity. Accordingly, molecular mechanisms of cancer cell fusion or specific molecular patterns of cancer hybrid cells may provide preferred targets for tumor-therapeutic interventions of identified fusion-based tumors.

## Figures and Tables

**Table 1:** Identification of tumor hybrid cells in human cancers.

Tumor Type	Normal Cells	Marker	Reference
Breast cancer	Macrophages	CD163, MAC387, DAP12	[182,184,197]
Colon/colorectal cancer	Macrophages	CD163, MAC387, DAP12, CD45, CD14, Cytokeratin	[183,192,197]
Epithelial ovarian carcinoma	BMDCs	CD45, CXCR4	[138]
Melanoma	Macrophages	CD14, CD45, Cytokeratin	[192]
	Stromal cells	BRAF (V600E) mutation	[190]
	BMDCs	STR analysis *	[185,186]
Multiple myeloma	Osteoclasts	Myeloma specific translocations	[187,188,219]
Non-small cell lung cancer	Macrophages	CD14, CD45, Cytokeratin, EpCAM	[194]
Pancreatic ductal adenocarcinoma	Macrophages	CD45, Cytokeratin	[191]
	BMDCs	CD45, EpCAM,	[164]
		Y chromosome *	
Renal cell carcinoma	BMDCs	blood group alleles *	[189]
	BMDCs	Y chromosome *	[196]

* Cancer patients with a BMT history.

## Abbreviations

BMT	bone marrow transplantation
CAFs	cancer-associated fibroblasts
HGT/LGT	horizontal gene transfer/lateral gene transfer
HST	heterokaryon-to-synkaryon transition
MSC	mesenchymal stroma-/stem-like cells
NSCLC	non-small cell lung cancer
PHPP	pre-hybrid preparation process
PHSP	post-hybrid selection process
PR	ploidy reductions
PS	phosphatidylserine
TNF-α	tumor necrosis factor-α
